# Protocol for selective *in vivo* labeling of bone marrow plasma cells by intrafemoral antibody injection in mice

**DOI:** 10.1016/j.xpro.2026.104750

**Published:** 2026-07-29

**Authors:** Zhuoyun Tong, Yiming Zhu, Haimei Lv, Shuangshuang Liu, Chunliang Xu

**Affiliations:** 1 Key Laboratory for Stem Cells and Tissue Engineering, Ministry of Education, Zhongshan School of Medicine, Sun Yat-sen University, Guangzhou, China; 2 Department of Histology and Embryology, Zhongshan School of Medicine, Sun Yat-sen University, Guangzhou, China; 3 Department of Pathology, School of Basic Medical Sciences, Xinjiang Medical University, Urumqi, China

**Keywords:** Cell Biology, Cell isolation, Flow Cytometry, Immunology, Model Organisms

## Abstract

Plasma cells are essential for long-term humoral immunity and are enriched in the bone marrow. Here, we present a protocol for selective labeling of plasma cells within a single mouse femur, using an intrafemoral antibody injection approach. We describe steps for the direct injection of fluorochrome-conjugated antibodies into the medullary cavity. We then detail procedures for bone marrow cell collection and analysis by flow cytometry. This protocol enables investigation of bone marrow plasma cell retention, redistribution, and mobilization.

For complete details on the use and execution of this protocol, please refer to Zhu et al.^1^

## Highlights

• Steps for intrafemoral antibody injection into mouse femoral bone marrow
• Steps for flow cytometric identification of antibody-labeled plasma cells
• Localized intrafemoral antibody injection labels bone marrow plasma cells *in vivo*
• Effective identification of plasma cells involved in bone marrow mobilization

## Abstract (editor highlights)

Publisher’s note: Undertaking any experimental protocol requires adherence to local institutional guidelines for laboratory safety and ethics.

## Graphical abstract

**Figure:**
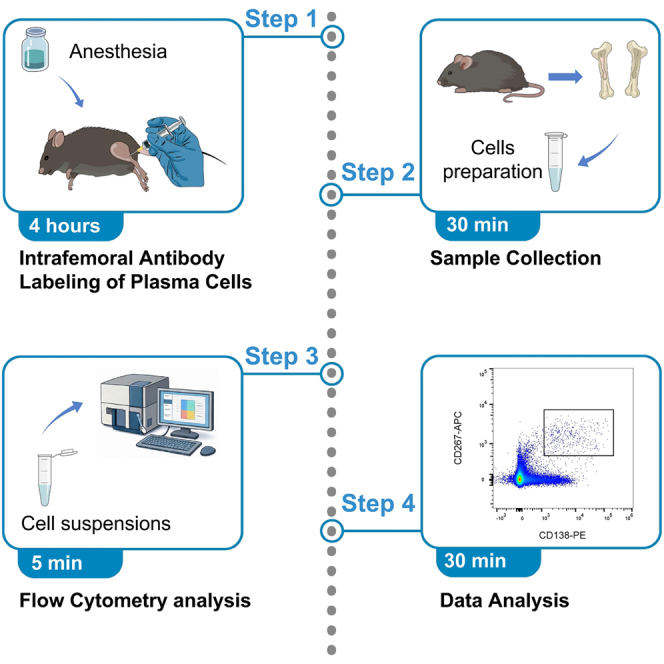

## Before you begin

Plasma cells are specialized terminally differentiated B cells, which constitute the major antibody-secreting effector population of the humoral immune system.^2^^,^^3^ Upon antigen stimulation, activated B cells undergo differentiation into plasma cells, which produce antigen-specific antibodies and provide essential protection against pathogens.^4^^,^^5^^,^^6^ Although plasma cells comprise populations with distinct lifespans and anatomical localizations,^7^^,^^8^^,^^9^ long-lived plasma cells (LLPCs) are particularly important for sustained humoral immunological memory because they continuously secrete protective antibodies over prolonged periods.^2^^,^^10^^,^^11^^,^^12^ The long-term survival of LLPCs depends on specialized bone marrow niches^13^ that provide essential signals for their retention, maintenance, and function.^14^^,^^15^^,^^16^ Given the important role of their residence in the bone marrow, reliable approaches to identify and study their migration, retention, and mobilization are crucial.^17^

This protocol describes a spatially restricted in vivo labeling approach for bone marrow plasma cells in mice. Labeling antibodies are directly injected into a single femoral cavity, enabling subsequent tracking of labeled plasma cells in non-injected bones, peripheral blood, or other tissues. The method labels plasma cells selectively in the injected femur, with minimal labeling in non-injected bones and has been validated in adult C57BL/6J mice.^1^ This method could potentially be adapted for in vivo labeling of other bone marrow cell populations by targeting their specific surface markers. Before starting the experiment, prepare 6- to 8-weeks-old specific pathogen–free (SPF) male C57BL/6J mice weighing 18–25 g. Confirm that all animals are in good health and show no obvious signs of stress. Prepare antibodies for plasma cell labeling (e.g., anti-CD138 and anti-CD267)^14^^,^^17^^,^^18^ and dilute them to the appropriate working concentrations according to experimental design. Place the mouse on the sterile drape and anesthetize the mouse using isoflurane gas for intrafemoral antibody injection.

### Innovation

Current approaches, such as flow cytometry and ELISpot, enable quantification analysis of plasma cells following ex vivo isolation of bone marrow samples.^19^^,^^20^ However, they cannot perform local in vivo labeling or fate tracking of endogenous bone marrow plasma cells. Consequently, they cannot distinguish whether a change in plasma cell numbers results from mobilization into circulation or local depletion. To address this, we describe an intrafemoral in situ labeling strategy that selectively labels plasma cells within a single bone, providing a basis for future migration-tracking studies. Compared with conventional methods using genetically engineered mice or antibody staining after cell isolation, this protocol employs direct intrafemoral injection to restrict antibody exposure to a defined local bone marrow space, achieving more selective labeling of endogenous plasma cells.^21^^,^^22^

The bone marrow cavity constitutes a relatively confined anatomical compartment, which permits efficient local distribution of antibodies with a minimal injection volume.^23^ By directly administering antibodies into the femoral bone marrow cavity, this protocol enables selective labeling of bone marrow-resident plasma cells, while minimizing labeling of plasma cells located in other bones and peripheral immune tissues, such as the spleen and lymph nodes. This spatially restricted labeling strategy provides a simple and reproducible approach to distinguish specific bone marrow-resident plasma cells from other plasma cell populations.

In addition, this strategy could potentially be extended to other bone marrow cell populations by using antibodies targeting surface markers. The key advantage of this intrafemoral in situ labeling approach is that it allows researchers to distinguish changes in plasma cell numbers due to local depletion from those due to mobilization or redistribution. By specifically labeling plasma cells in a single bone, one can monitor whether labeled cells subsequently appear in other bones or in peripheral blood under experimental conditions (e.g., fasting, inflammation, or pharmacological intervention). This protocol has been successfully applied to study fasting-induced plasma cell mobilization^1^ and can be adapted for various other contexts.

### Institutional permissions

All mouse experiments were approved by the Animal Ethics Committee of Zhongshan School of Medicine, Sun Yat-sen University (approval number: 2024003240) and were conducted in accordance with institutional and national guidelines for the care and use of laboratory animals. Researchers should obtain appropriate animal ethics approval from their own institutions before using this protocol.

## Key resources table

**Table:** 

REAGENT or RESOURCE	SOURCE	IDENTIFIER
**Antibodies**		

Anti-mouse CD138-PE (Dilution 1/10)	BioLegend	142504; RRID: AB_10916119
Anti-mouse CD267-APC (Dilution 1/10)	eBioscience	17-5942-82; RRID: AB_842758

**Chemicals, peptides, and recombinant proteins**		

DAPI	Sigma-Aldrich	Cat# D9542-5MG
Bovine serum albumin (BSA)	ABCone	Cat# A23088-100G
NaCl	Sigma-Aldrich	Cat# S9888-500G
KCl	Sigma-Aldrich	Cat# P9541-500G
Na_2_HPO_4_	Sigma-Aldrich	Cat# S9763-500G
KH_2_PO_4_	Sigma-Aldrich	Cat# P0062-500G
Na_2_EDTA·2 H_2_O	Sigma-Aldrich	Cat# E5134-50G
NH_4_Cl	Sigma-Aldrich	Cat# 254134-100G
KHCO_3_	Sangon	Cat# A501195-0500
Buprenorphine	Qinghai Pharmaceutical	Cat# H10940181
Sterile saline (0.9% NaCl)	ServiceBio	Cat# G4702
Carprofen	Aladdin	Cat# C153919
Isoflurane	RWD Life Science	Cat# R510-22
Plerixafor (AMD3100) 8HCl	Selleck	Cat# S3013

**Experimental models: Organisms/strains**		

Mouse: C57BL/6J (Male, 6-8 weeks old)	GemPharmatech	N/A

**Software and algorithms**		

FlowJo (v10.8.1)	BD Biosciences	https://www.flowjo.com/solutions/flowjo/downloads
Attune™ NxT Software (v6.2.2)	Thermo Fisher Scientific	Cat# A25554
GraphPad Prism (v9.0)	GraphPad	https://www.graphpad.com

**Other**		

Attune™ NxT Flow Cytometer	Thermo Fisher Scientific	Cat# A24858
Centrifuge	Eppendorf	Cat# 5810 R
Compact small animal anesthesia machine	RWD Life Science	Cat# R500IP
Anesthesia air pump	RWD Life Science	Cat# R510-30
Gas filter canister	RWD Life Science	Cat# R510-31S
Low stress anesthesia induction chamber	RWD Life Science	Cat# V105
Cone mask with tubing	RWD Life Science	Cat# 68681
Induction chamber-mouse	RWD Life Science	Cat# V100
Depilatory paste	Veet	Cat# 3282427
1 mL disposable plastic syringe (needle-free)	Cangzhou Quansheng	Cat# 20240405
Disposable sterile syringes	MINANK	Cat# 0.8 × 35TWLB (21 G)
Disposable sterile insulin syringe (with 30 G needle)	Yeso-med	Cat# 0.3 × 12.7 mmRWLB
Disposable sterile syringes	MINANK	Cat# 0.6×25TWLB (23 G)
Electric shaver	Kerong Biotechnology	Cat# XS-0055
Sterile cloth	Zhonggan Medical	Cat# MF1035
Adhesive tape	Xinhua Medical	Cat# 695182779667XH
Cotton swabs	Haishi Hainuo Medical Technology	Cat# 677831206450
Puralube ophthalmic ointment	Puralube®	Cat# 17033-211-38
Heating pad	Yuyan instrument	Cat# HP-4M
Forceps	LIGE Technology	Cat# LG01-107-4I
Sterile scissors	LIGE Technology	Cat# LG01-105-8M
1.5 mL Microcentrifuge tube	NEST Biotechnology	Cat# 615001
Povidone-iodine (10%)	Haishi Hainuo Medical Technology	Cat# A2045
BD Vacutainer® EDTA Tubes	BD	Cat# 367841-2 mL

## Materials and equipment

**Table:** 10 × PBS (Phosphate Buffered Saline, pH 7.4)

Reagent	Final concentration	Amount
NaCl	1.37 M	80.06 g
KCl	27 mM	2.01 g
Na_2_HPO_4_	100 mM	14.20 g
KH_2_PO_4_	18 mM	2.45 g
ddH_2_O	N/A	Up to 1L
**Total**	**N/A**	**1 L**

Dissolve the reagents in approximately 500 mL ddH_2_O. Adjust the final volume to 1 L with additional ddH_2_O. Store at 4°C for up to 3 months.

**Table:** 1 × PBS

Reagent	Final concentration	Amount
10 × PBS	N/A	100 mL
ddH_2_O	N/A	900 mL
**Total**	**N/A**	**1 L**

Store at 4°C for up to 1 month.

**Table:** 0.5 M EDTA (Ethylenediaminetetraacetic acid)

Reagent	Final concentration	Amount
Na_2_EDTA·2 H_2_O	0.5 M	186.1 g
ddH_2_O	N/A	Up to 1L
**Total**	**N/A**	**1 L**

Dissolve the reagents in approximately 800 mL of ddH_2_O. Adjust the pH to 8.0 with NaOH, then adjust the final volume to 1 L with additional ddH_2_O. Store at 4°C for up to 3 months.

**Table:** 10 × PEB (PBS with EDTA and BSA) Buffer

Reagent	Final concentration	Amount
10 × PBS	N/A	100 mL
BSA	5% (m/v)	50 g
EDTA, 0.5 M, pH 8.0	20 mM	40 mL
ddH_2_O	N/A	Up to 1L
**Total**	**N/A**	**1 L**

Dissolve the reagents in approximately 500 mL ddH_2_O. Adjust the final volume to 1 L with additional ddH_2_O. Store at 4°C for up to 3 months.

**Table:** 1 × PEB Buffer

Reagent	Final concentration	Amount
10 × PEB	N/A	100 mL
ddH_2_O	N/A	900 mL
**Total**	**N/A**	**1 L**

Store at 4°C for up to 1 month.

**Table:** 10 × RBC Lysis Buffer

Reagent	Final concentration	Amount
NH_4_Cl	8.26% (m/v)	82.6 g
KHCO_3_	1% (m/v)	10.0 g
EDTA, 0.5 M, pH8.0	0.5 mM	1 mL
ddH_2_O	N/A	Up to 1L
**Total**	**N/A**	**1 L**

Dissolve the following reagents in approximately 800 mL of ddH_2_O. Adjust the pH to 7.2–7.4 using 1 N HCl, then adjust the final volume to 1 L with ddH_2_O. Store at 4°C for up to 3 months.

**Table:** 1 × RBC Lysis Buffer

Reagent	Final concentration	Amount
10 × RBC lysis Buffer	N/A	100 mL
ddH_2_O	N/A	900 mL
**Total**	**N/A**	**1 L**

Store at 4°C for up to 1 month. Other commercially available RBC lysis Buffer can also be used.

**Table:** DAPI stock solution

Reagent	Final concentration	Amount
DAPI	1 mg/mL	1 mg
ddH_2_O	N/A	1 mL
**Total**	**N/A**	1 mL

Store in the dark in foil-wrapped tubes at −20°C.

**Table:** DAPI working solution

Reagent	Final concentration	Amount
DAPI stock solution	0.2 μg/mL	10 μL
1 × PEB buffer	N/A	50 mL
**Total**	**N/A**	50 mL

Prepare fresh before use and keep protected from light until use.

**Table:** AMD3100 stock solution

Reagent	Final concentration	Amount
Plerixafor (AMD3100) 8HCl	10 mg/mL	10 mg
Sterile 1 × PBS buffer	N/A	1 mL
**Total**	**N/A**	**1 mL**

Aliquot 100 μL per tube in 200 μL tubes and keep at −80°C.

**Table:** AMD 3100 working solution

Reagent	Final concentration	Amount
AMD3100 stock solution	1 mg/mL	100 μL
Sterile 1 × PBS buffer	N/A	Up to 1 mL
**Total**	**N/A**	**1 mL**

Prepare fresh before use and keep protected from light until use.

## Step-by-step method details

### Preparation for intrafemoral injection

**Timing: 10–15 min**

1. Prepare the labeling antibody solution in PEB buffer with each antibody at a final concentration of 20 μg/mL (Working dilution for antibodies: 1:10).

***Note:*** Antibody solutions should be freshly prepared to avoid loss of antibody activity during prolonged storage. Then keep it on the ice and protect from light until use.

2. Prepare a sterile 0.3 mL 30 G insulin syringe.
3. Prepare 10% povidone-iodine by using the undiluted commercial stock solution and establish an aseptic working area before the procedure.

**CRITICAL:** All materials for injection must be sterile before use. Once the antibody is aspirated, injection should be performed promptly to minimize volume instability caused by repeated handling.

### Preparation for bone marrow and cell collection

**Timing: 15–20 min**

4. Prepare PEB (PBS with EDTA and BSA) buffer as described in the materials and equipment.

***Note:*** PEB buffer used for cell isolation and analysis should be kept cold to preserve cell viability.

5. Prepare RBC lysis buffer as described in the materials and equipment section.

***Note:*** RBC lysis buffer should be kept cold.

***Note:*** Alternatively, a commercially available RBC lysis buffer (e.g., Thermo Fisher #00-4333-57) can be used according to the manufacturer’s instructions.

6. Prepare sterile dissection instruments and 1 mL syringe with 21 G needle.
7. Pre-cool centrifuge to 4°C and prepare one microcentrifuge tube per sample for bone marrow cell suspensions.
8. Prepare fresh viability dye by diluting DAPI stock solution (1 mg/mL) 1:5000 in PEB buffer before flow cytometry, resulting in a concentration of 0.2 μg/mL.

***Note:*** In live, non-permeabilized cells, DAPI is usually excluded by intact membranes, so live cells appear DAPI-negative.

***Note:*** The diluted DAPI working solution should be freshly prepared before use and kept on ice protected from light.

**CRITICAL:** EDTA-containing buffers are required to prevent cell aggregation during bone marrow flushing and antibody staining.

### Intrafemoral antibody injection

**Timing: 5–10 min per mouse**

**Figure 1:**
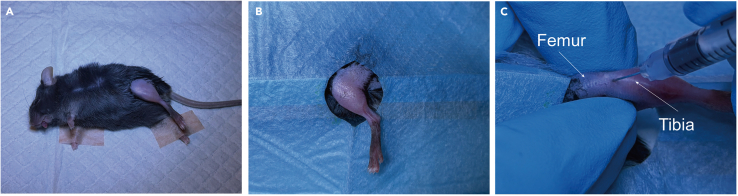
Intrafemoral injection procedure in mice (A) Lateral recumbency of the anesthetized mouse on a sterile drape, with the shaved hind limb secured using surgical tape. (B) Exposure of the knee region for identification of the distal femur and proximal tibia, followed by skin disinfection with povidone-iodine. (C) Needle insertion through the knee joint into the distal femur for delivery into the femoral medullary cavity. Arrows indicate the femur and tibia.

This step describes intrafemoral antibody injection for in situ labeling of bone marrow plasma cells in mice (Figure 1).

9. Induce and maintain anesthesia in mice using isoflurane gas.
  a. Place the mice in an induction chamber and induce anesthesia using 3%–4% (v/v) isoflurane at an oxygen flow rate of 0.8–1.0 L/min.
  b. After approximately 1–2 min, the righting reflex disappears, indicating that anesthesia induction is complete.
  c. Quickly transfer the mouse to a nose cone and maintain anesthesia using 1.5%–2% (v/v) isoflurane at an oxygen flow rate of 0.3–0.5 L/min.

***Note:*** Before injection, ensure the mice are fully anesthetized to avoid movement during the procedure and to reduce the risk of inaccurate injection or bone fracture.

***Note:*** Determine whether the anesthesia state in mice is adequate by observing the disappearance of relevant reflexes, such as pedal withdrawal reflex.

10. After confirming adequate anesthesia, apply eye ointment to prevent dry eyes during anesthesia.
11. Skin preparation using an electric shaver and depilatory cream.
  a. Remove hair around the knee joint using an electric shaver.
  b. Leave the depilatory cream in place for 5–10 s,
  c. Remove the depilatory cream with a sterile cotton ball.
  d. Rinse the skin with saline to minimize irritation.
12. Place the mouse on a heating pad covered with sterile cloth. Secure the limbs to the surgical platform with adhesive tape to fully expose the hind limb (Figures 1A and 1B**)**.
13. Disinfect the surgical site three times using cotton swabs soaked in povidone-iodine.
14. Use the thumb and index finger of one hand to stabilize the hind limb and gently flex the knee joint to expose the distal femur.
15. Maintain stabilization of the femoral shaft and identify the injection site at the distal femoral articular surface.
16. Insert the needle into the femoral cortex with the other hand, and advance it with gentle clockwise and counterclockwise rotation while sensing resistance from the cortical bone (Figure 1C**)**.

***Note:*** For intrafemoral injection in mice, a needle no larger than 30 G is recommended to minimize mechanical damage to bone tissue.^22^^,^^24^

***Note:*** The insertion depth is typically about 0.2–0.3 cm. When a sudden loss of resistance is felt, stop advancing immediately to avoid excessive penetration and mechanical damage.

17. Slowly inject the antibody solution while gently rotating the syringe.

***Note:*** The injection volume is 10 μL per injection. Repeated injections or aspiration of excessive volumes at one time may increase plunger resistance and compromise precise volume control.^23^

***Note:*** Inject slowly and continuously to facilitate even antibody distribution within the bone marrow cavity and reduce the risk of leakage or injection failure.

18. After injection, apply gentle pressure with a sterile cotton swab for approximately 30 s to minimize bleeding and disinfect the injection site again with povidone-iodine.
19. Administer pre-emptive analgesia by subcutaneously injecting a mixture of buprenorphine (0.1 mg/kg) and carprofen (5 mg/kg).
20. To prevent dehydration, administer 0.5 mL sterile saline subcutaneously to each mouse.
21. Place the mice on a heating pad until they are fully recovered from anesthesia and monitor them continuously for signs of pain or distress, such as paw licking or reduced mobility.

***Note:*** Although rare, any mice exhibiting severe adverse events should be euthanized immediately. These include persistent bleeding that cannot be controlled by gentle pressure within 1–2 min, obvious abnormal limb angulation, abnormal mobility or crepitus suggestive of fracture, complete inability to bear weight or continuous dragging of the affected limb, or persistent signs of severe pain or distress, including self-biting, self-licking, curling up, or rapid breathing, especially if not relieved by analgesic intervention.

**CRITICAL:** Do not advance the needle further after entering the bone marrow cavity. Excessive penetration may damage the marrow cavity and alter antibody distribution, reducing labeling efficiency and reproducibility.

### Post-injection recovery and completion of bone marrow plasma cell labeling

**Timing: 4 h**

This step describes post-injection recovery monitoring and the timing required for antibody distribution and completion of plasma cell labeling in the bone marrow.

22. Place injected mice in a clean, warm recovery environment until they regain full consciousness and spontaneous activity.
23. Monitor the general condition of mice during recovery, including locomotion, feeding behavior, and signs of persistent pain or abnormal behavior.
24. Maintain mice for 4 h after intrafemoral injection.^1^
25. At 4 h post-injection, proceed to tissue collection for flow cytometry analyses.

**CRITICAL:** Prolonged intervals between injection and tissue collection may lead to antibody leakage into the peripheral circulation via bone marrow sinusoids, increasing the risk of labeling non-bone marrow-resident cells.

### Bone marrow cell collection

**Timing: 30 min per mouse**

**Figure 2:**
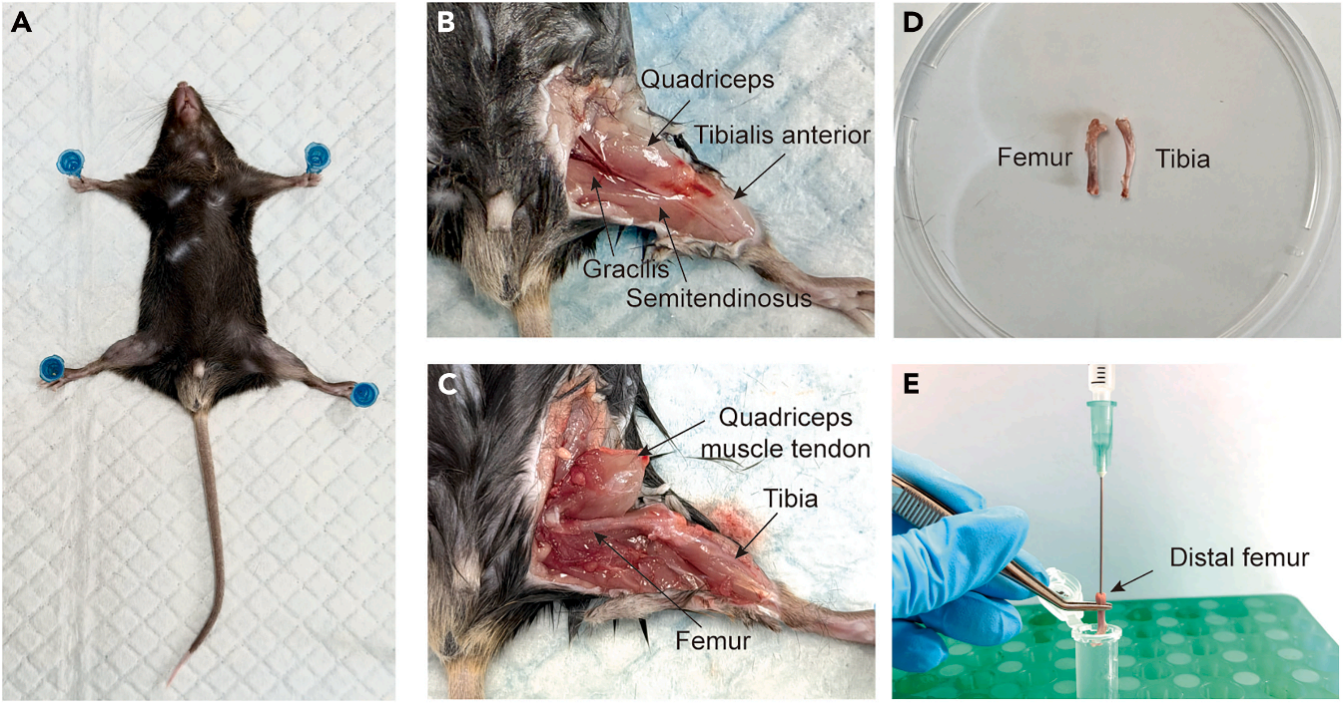
Dissection of mouse femur and tibia and collection of bone marrow cells (A) Mouse is euthanized by overdose anesthesia, placed in a supine position, and secured on a dissection board with pins. (B) An incision is made in the inguinal region to expose the hindlimb musculature, including the quadriceps, tibialis anterior, and semitendinosus muscles. (C) Surrounding muscles and connective tissues are carefully removed to expose the femur and tibia. (D) The femur and tibia are dissected free from adjacent tissues and transferred to a sterile dish. (E) Bone marrow cells are flushed from distal femur with a syringe containing PEB buffer to obtain a bone marrow cell suspension. The tibia is processed in the same manner.

This step describes the isolation of bone marrow cells following intrafemoral antibody injection and their preparation for flow cytometric analysis (Figure 2). The method of bone marrow cell collection has been validated in C57BL/6J mice in our previous studies.^1^^,^^16^^,^^25^

26. At approximately 4 h after intrafemoral injection, euthanize the mouse by carbon dioxide inhalation and place it in a supine position (Figure 2A).
27. Make an incision along the inguinal region and gently separate the skin to expose the underlying muscle tissue (Figure 2B).
28. Transect the quadriceps tendon at the knee joint and carefully separate the quadriceps muscle. On the dorsal side, dissect the gastrocnemius muscle toward the semitendinosus muscle to expose the hip joint (Figure 2C).
29. Femoral collection: Disarticulate both femoral heads and remove surrounding muscle tissue. Separate the distal femur from the knee joint and cut off the femoral head to expose the bone marrow cavity (Figure 2D).
30. Tibial collection: Disarticulate both tibiae from the ankle joint. Remove surrounding muscle and connective tissue. Trim the proximal tibial plateau and distal tibial end using sterile scissors to expose the tibial bone marrow cavity (Figure 2D).
31. Draw up 1 mL of PEB buffer using a 1 mL syringe with a 21 G needle, and flush the bone marrow cavity into a microcentrifuge tube (Figure 2E**)**.
32. Centrifuge the bone marrow cell suspension at 400 × *g* for 5 min at 4°C.
33. Carefully discard the supernatant and resuspend the cell pellet in 1 mL RBC lysis buffer. Incubate on ice for 5 min.
34. Quench the lysis reaction by adding 1 mL PEB buffer, then centrifuge again at 400 × *g* for 5 min at 4°C.
35. Discard the supernatant and resuspend the final cell pellet with 1 mL PEB buffer.

***Note:*** Typically, 150 μL of the cell suspension, containing approximately 2 × 10^6^ cells, is pre pared for flow cytometric analysis.

**Pause point:** After resuspension in PEB buffer, bone marrow cell suspensions can be kept on ice and protected from light for up to 1 h before flow cytometric analysis. Avoid longer storage, as this may reduce cell viability and affect labeling quality.

36. Before flow cytometric analysis, mix the DAPI working solution with 150 μL of bone marrow cell suspension at a 1:1 ratio to exclude dead cells.

***Note:*** The opposite side femur and bilateral tibia are used as the non-injected control bone for assessing whether antibody labeling is restricted to the injected femur.

**CRITICAL:** Excessive mechanical force during bone marrow flushing or prolonged RBC lysis may reduce cell viability and compromise subsequent flow cytometry results.

### Peripheral blood cell collection

**Timing: 10–15 min per mouse**

37. Peripheral blood collection.
  a. Anesthetize the mouse with 3%–4% (v/v) isoflurane at an oxygen flow rate of 0.8–1.0 L/min.
  b. Confirm adequate anesthesia by the absence of a pedal withdrawal reflex.
  c. Place the mouse in a supine position on a clean working surface.
  d. Collect terminal blood by cardiac puncture using a 1 mL sterile syringe fitted with a 23G needle.
38. Transfer the collected blood immediately into an anticoagulant-treated EDTA tube and gently mix by inversion to prevent clotting.
39. After blood collection, euthanize the mouse immediately by carbon dioxide inhalation or cervical dislocation.
40. Add 1 mL RBC lysis buffer and incubate on ice for 5 min.
41. Centrifuge at 400 × *g* for 5 min at 4°C and then carefully discard the supernatant.
42. Repeat steps 40 and 41 three times to ensure complete lysis of red blood cells.
43. Quench the lysis reaction by adding 1 mL PEB buffer, then centrifuge again at 400 × *g* for 5 min at 4°C.
44. Discard the supernatant and resuspend the final cell pellet with 1 mL PEB buffer. Typically, 200 μL of the cell suspension, containing approximately 5 × 10^5^ cells, is prepared for flow cytometric analysis.

**Pause point:** After RBC lysis and resuspension in PEB buffer, peripheral blood cell suspensions can be kept on ice for up to 1 h before flow cytometric analysis. Keep samples protected from light and proceed to acquisition as soon as possible.

45. Before flow cytometric analysis, mix the DAPI working solution with 200 μL of cell suspension at a 1:1 ratio to exclude dead cells.

***Note:*** Keep the samples on ice and protected from light until flow cytometry acquisition.

### Flow cytometric analysis

**Timing: 30 min**

**Figure 3:**
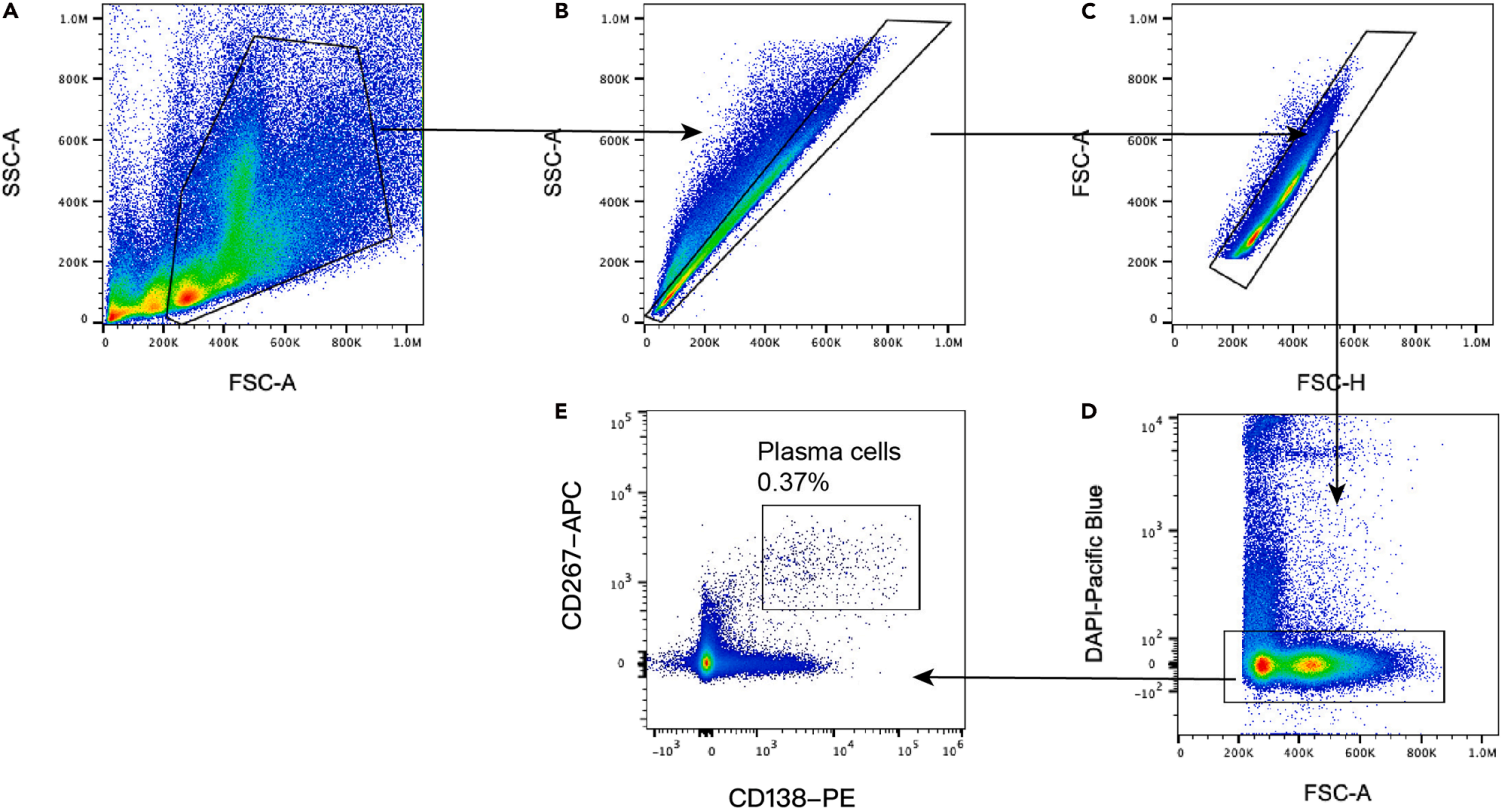
Flow cytometry gating strategy for identifying antibody-labeled plasma cells in bone marrow (A) Bone marrow cells are first gated based on forward scatter (FSC-A) and side scatter (SSC-A) to exclude debris. (B and C) Doublets are excluded by sequential gating singlet cells using SSC-A versus SSC-H and FSC-A versus FSC-H. (D) Dead cells are excluded by DAPI staining to identify the live-cell population. (E) Plasma cells are defined as CD138^+^ CD267^+^ cells.

This step describes the gating strategy for plasma cells analyzed by flow cytometry. Gates are applied as shown in Figure 3.

46. Controls for flow cytometric analysis.
  Before performing flow cytometric analysis, it is necessary to set up appropriate parameters, including negative controls, compensation controls, and FMO controls. These controls are required to properly define positive populations and compensate for spectral overlap.
  a. Negative (uninjected) control.
    Process bone marrow cells from the non-injected contralateral femur identically and use them to establish the background fluorescence threshold for CD138-PE and CD267-APC.
  b. Compensation controls.
    Prepare single-color controls by staining bone marrow cells from an uninjected mouse with either CD138-PE or CD267-APC ex vivo. Use these controls to calculate the compensation matrix.
  c. FMO controls.
    Prepare fluorescence-minus-one controls (e.g., all antibodies except CD138-PE, or all except CD267-APC) by ex vivo staining of uninjected bone marrow cells to assess spillover-induced false positivity.
    ***Note:*** Cells with fluorescence intensity above the background level are defined as positive.
47. Sample acquisition.

Acquire the sample on the flow cytometer. Subsequently, identify CD138^+^ CD267^+^ plasma cells based on the gating strategy described in the following steps.

48. Gating strategy.
  a. Exclusion of debris.
    Gate cells based on forward scatter (FSC) and side scatter (SSC) parameters to exclude debris and very small particles (Figure 3A**)**.
  b. Singlet discrimination.
    Exclude doublets and cell aggregates by gating on FSC-A versus FSC-H (or FSC-W), retaining only single-cell events (Figures 3B and 3C**)**.
  c. Live cell gating.
    Exclude dead cells based on viability dye staining and retain viability dye-negative cells (Figure 3D**)**.
  d. Identification of plasma cells.
    Define plasma cells as CD138^+^ CD267^+^ cells within the viable singlet population (Figure 3E**)**.
49. Assessment of in vivo antibody labeling.

Assess antibody labeling efficiency by measuring the percentage of antibody-positive cells within the CD138^+^ CD267^+^ plasma cell gate.

## Expected outcomes

The primary utility of this protocol lies not merely in quantifying labeled plasma cells within the injected femur, but in using the injected femur as a spatially restricted in vivo labeling site to track the subsequent retention, redistribution, or mobilization of labeled plasma cells outside the injection site, such as to non-injected bones or peripheral blood.Therefore, the expected outcomes can be used to validate the specificity of local labeling and the downstream application in detecting plasma cell mobilization.

**Figure 4:**
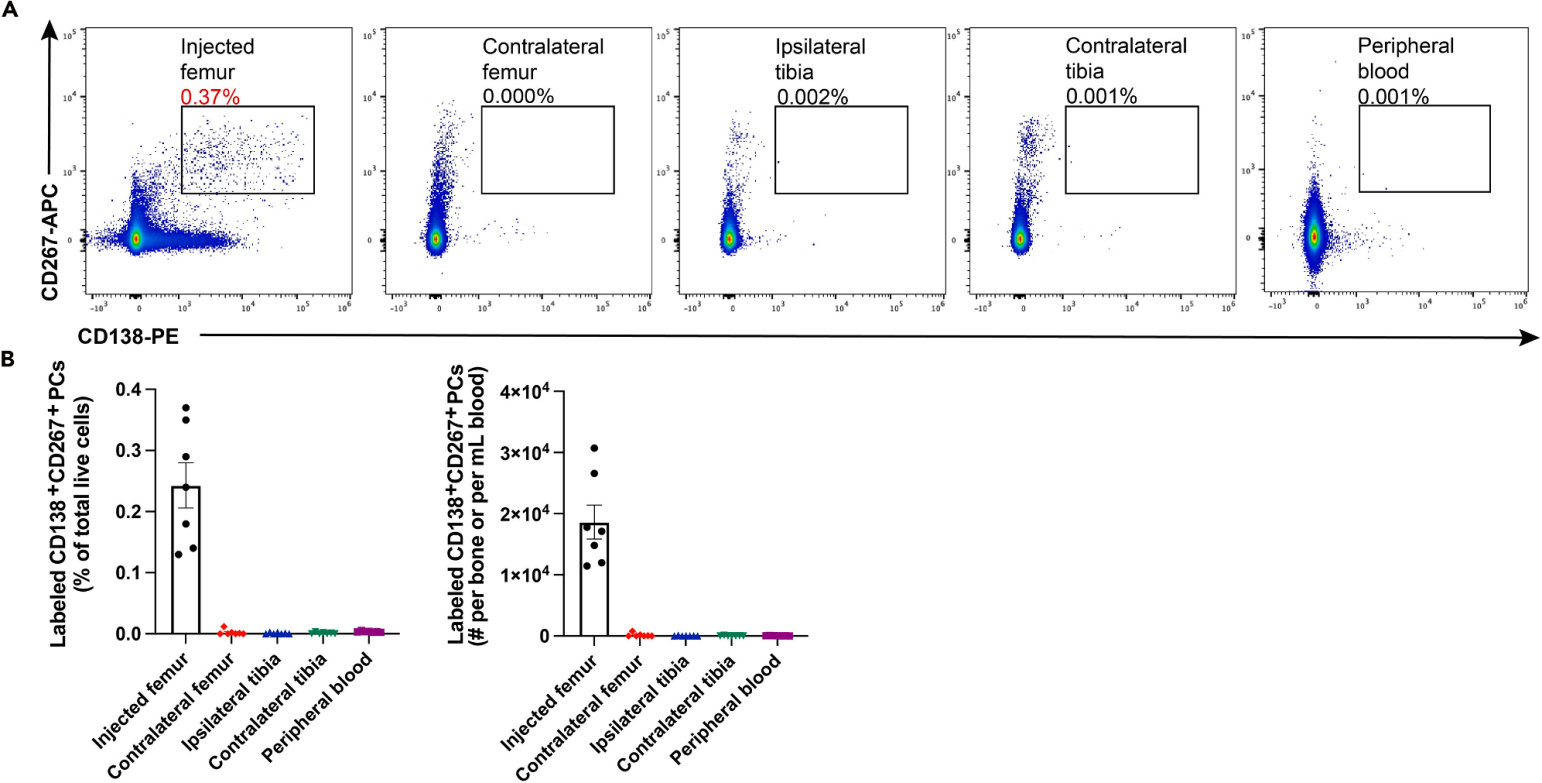
Selective labeling of plasma cells in the injected femur after intrafemoral antibody injection (A) Representative flow cytometry plots of cells isolated from the injected femur, contralateral femur, ipsilateral tibia, contralateral tibia, and peripheral blood in mice injected with plasma cell-specific antibodies. Gates indicate labeled CD138^+^ CD267^+^ plasma cells. (B) Quantification of the percentage and absolute number of labeled plasma cells in the injected femur, contralateral femur, ipsilateral tibia, contralateral tibia and peripheral blood (Data are shown as mean ± SEM; n=7 mice). Data are shown as mean ± S.E.M.

For validation of local labeling specificity, intrafemoral antibody injection should result in antibody labeling that is largely restricted to the injected femoral bone marrow cavity. At 4 h after injection of antibodies, flow cytometric analysis should show enrichment of labeled CD138^+^ CD267^+^ plasma cells in the injected femur (Figure 4). In our experiments, labeled CD138^+^ CD267^+^ plasma cells accounted for approximately 0.37% of total bone marrow cells in the injected femur, whereas few or no antibody-labeled cells were detected in the contralateral femur or in the ipsilateral and contralateral tibiae (Figures 4A and 4B). Under steady-state conditions, labeled CD138^+^ CD267^+^ plasma cells should also be minimal in peripheral blood. Detection of antibody-positive CD138^+^ CD267^+^ plasma cells in peripheral blood under experimental conditions may indicate mobilization or redistribution of labeled plasma cells from the injected femur. Successful labeling is indicated by clear enrichment of antibody-positive CD138^+^ CD267^+^ plasma cells in the injected femur, with minimal or undetectable labeling in non-injected bones and peripheral blood under steady-state conditions.

**Figure 5:**
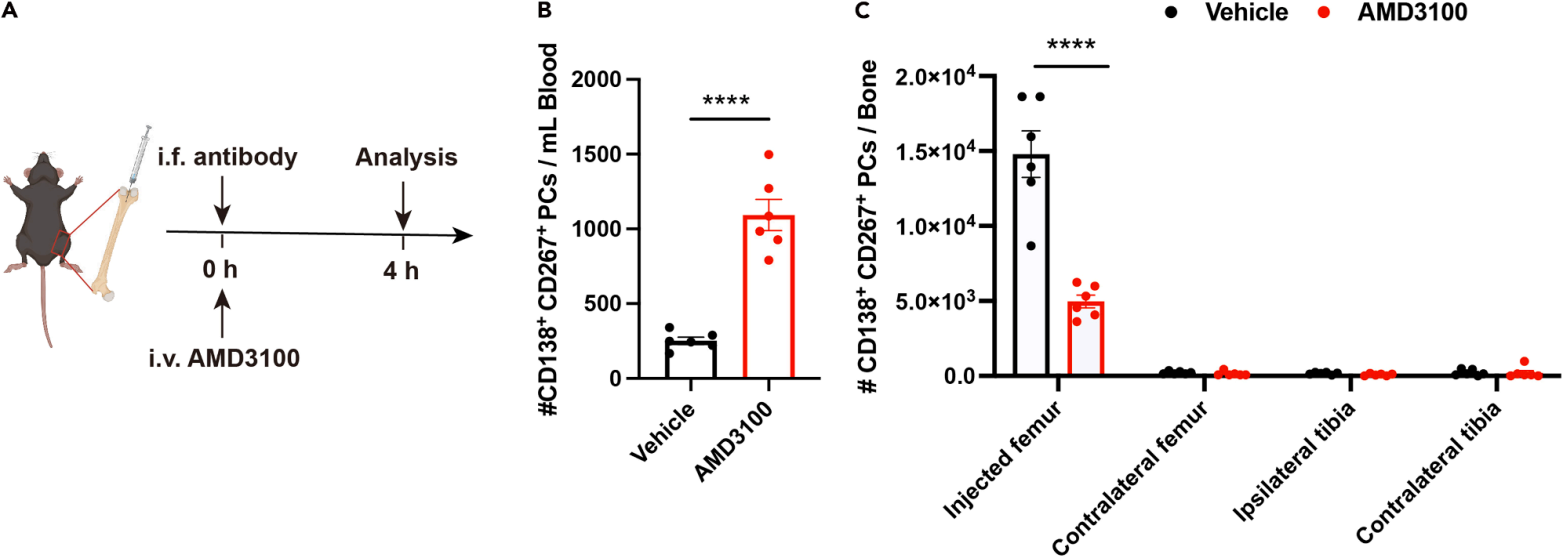
Validation of the protocol for plasma cell mobilization detection using AMD3100 (A) Experimental scheme for evaluating the mobilization of plasma cells from bone marrow into peripheral blood using AMD3100. (B) Number of labeled plasma cells in the blood of mice with or without AMD3100 treatment (n=6 mice/group). (C) Number of labeled plasma cells in different bones of mice with or without AMD3100 treatment (n=6 mice/group). Data are mean ± S.E.M. ∗∗∗∗*p* < 0.0001 by unpaired two-tailed Student's *t* test.

After local labeling specificity has been confirmed, this protocol can be used to assess whether labeled plasma cells are retained in the injected femur or mobilized to other compartments. Under steady-state or vehicle-treated conditions, labeled CD138^+^ CD267^+^ plasma cells should be rare in peripheral blood. As shown in the original study^1^ and in Figure 5, plasma cell mobilization can be detected using AMD3100 treatment. AMD3100 was prepared at 1 mg/mL in sterile PBS and administered subcutaneously at 5 μL/g body weight, corresponding to a dose of 5 mg/kg. Compared with vehicle-treated controls, AMD3100-treated mice showed an increased number of labeled CD138^+^ CD267^+^ cells in peripheral blood (Figures 5A and 5B). This increase was accompanied by a reduction in labeled plasma cells in the injected femur, supporting mobilization of locally labeled bone marrow plasma cells (Figure 5C).

Mice usually recover rapidly after intrafemoral injection when the injection volume, needle depth, and antibody exposure time are well controlled. Overt signs of pain, impaired mobility, or abnormal recovery should not be observed under optimized conditions.

## Limitations

This protocol relies on precise intrafemoral injection to achieve localized antibody labeling,^1^ and therefore requires technical proficiency. Improper control of needle depth, angle, or injection volume may result in failure to access the bone marrow cavity, antibody leakage, reduced labeling efficiency, or increased nonspecific labeling.

In addition, antibody retention within the bone marrow cavity is limited. Prolonged intervals between injection and tissue collection may allow antibody to enter the peripheral circulation, thereby reducing the specificity of bone marrow plasma cell labeling. The isotype, affinity, and in vivo stability of the antibody may also affect labeling efficiency and should be optimized for individual applications.

Furthermore, this protocol is primarily optimized for intrafemoral labeling in adult mouse femurs. For juvenile mice, disease models with altered bone density, or alternative skeletal sites, adjustments to needle size, injection angle, and injection volume may be required. One anatomical limitation of this approach is that the initial labeling event is restricted to a single femoral marrow cavity. Therefore, the labeled plasma cells in the injected femur may not fully represent plasma cells distributed throughout the skeleton, including the tibiae, humeri, vertebrae, and sternum. This protocol should thus be interpreted as a spatially restricted in vivo labeling approach for tracking locally labeled plasma cells, rather than as a method for assessing whole-skeleton plasma cell dynamics.

## Troubleshooting

### Problem 1

Weak or absent plasma cell labeling after injection (related to steps 6–17).

### Potential solution

Confirm successful entry into the bone marrow cavity. A clear cortical resistance followed by a sudden loss of resistance sensation should be felt during needle insertion. Avoid shallow insertion into cortical bone or excessive penetration through the marrow cavity. In addition, a fluorophore-conjugated pan-hematopoietic marker, such as anti-CD45, can be co-injected as an internal injection control and, when appropriate, used as a normalization reference. Efficient labeling of CD45^+^ bone marrow hematopoietic cells would indicate successful antibody delivery into the marrow cavity, whereas weak or absent CD45 labeling may suggest failed or insufficient delivery. CD45 labeling may also help account for variability in cell recovery across samples.

### Problem 2

High inter-animal variability in labeling efficiency (related to steps 16–17).

### Potential solution

Standardize mouse age, sex, and body weight. Use consistent anatomical landmarks at the distal femur near the knee joint for injection.

### Problem 3

Antibody leakage or visible fluid at the injection site (related to step 16–17).

### Potential solution

Inject 10 μL per femur. If leakage occurs, reduce the volume to 5–8 μL, and adjust the antibody concentration accordingly to maintaining the same delivered antibody dose is required. Perform slow, continuous injection with gentle syringe rotation to reduce local pressure.

### Problem 4

When the injection is not optimal, the labeled plasma cells detected in the peripheral blood may not represent true cell mobilization but passive antibody leakage (related to step 16–17).

### Potential solution

Use non-mobilized control mice and AMD3100-treated mice as negative and positive mobilization controls, respectively. Under steady-state conditions, labeled CD138^+^CD267^+^ plasma cells should be extremely rare in peripheral blood, because bone marrow-resident plasma cells rarely enter the circulation. Significant peripheral labeling in non-mobilized controls may suggest antibody leakage. By contrast, true mobilization is supported when labeled plasma cells increase in peripheral blood specifically under the mobilizing condition, or after treatment with the CXCR4 antagonist AMD3100, compared with vehicle controls, while antibody labeling remains largely restricted to the injected femur. To reduce nonspecific leakage, shorten the antibody exposure time, verify the injection volume, and avoid shallow cortical injection or excessive needle penetration through the marrow cavity.

### Problem 5

Low cell viability or poor cell recovery after bone marrow collection (related to steps 31–45).

### Potential solution

• Optimize bone marrow flushing. Use a 21 G needle and apply gentle, steady pressure when flushing the bone marrow cavity with PEB buffer. Avoid vigorous or repeated flushing, as excessive mechanical force may damage cells.
• Limit RBC lysis time. Incubate the cell pellet in RBC lysis buffer for no more than 5 min on ice, as prolonged lysis can compromise the viability of nucleated cells.
• Keep all buffers cold. Use ice-cold PEB buffer and RBC lysis buffer throughout the procedure. Warm buffers may accelerate cell death and reduce viability.
• Use EDTA-containing buffer. PEB buffer contains EDTA to prevent cell clumping; ensure that the buffer is correctly prepared and kept on ice throughout the procedure.
• Centrifuge at appropriate speed and temperature. Centrifuge samples at 400 × *g* for 5 min at 4°C. Higher centrifugation speeds or room-temperature processing may increase cell loss or damage.

## Resource availability

### Lead contact

Further information and requests for resources and reagents should be directed to and will be fulfilled by the lead contact, Chunliang Xu (xuchliang@mail.sysu.edu.cn).

### Technical contact

Technical questions on executing this protocol should be directed to and will be answered by the technical contact, Chunliang Xu (xuchliang@mail.sysu.edu.cn).

### Materials availability

This study did not generate any new unique reagents. Requests for additional materials should be directed to the lead contact and will be considered on a case-by-case basis.

### Data and code availability

All datasets generated or analyzed in this study are included in the published article. Requests for additional data or materials should be directed to the lead contact. This protocol does not report original code.

## Acknowledgments

We thank the Core Facilities for Medical Sciences at the Zhongshan School of Medicine for assistance with flow cytometric analysis and the Laboratory Animal Center of Sun Yat-Sen University for technical assistance. This study was supported by grants from the 10.13039/501100012166National Key R&D Program of China (2022YFA1105000 and 2022YFA1104100), the 10.13039/100014718National Natural Science Foundation of China (82271783), the 10.13039/501100021171Guangdong Basic and Applied Basic Research Foundation (2023A1515012294), and the Guangzhou Basic and Applied Basic Research Foundation (2024A04J6379) to C.X.

## Author contributions

C.X. and Y.Z. conceived the study. Z.T., H.L., and S.L. performed the experiments. All authors contributed to the manuscript preparation and approved the final manuscript.

## Declaration of interests

The authors declare no competing interests.
